# Endothelin-2 from Keratinocytes and its Association with Itch in Skin Diseases

**DOI:** 10.1016/j.jid.2025.07.030

**Published:** 2025-08-22

**Authors:** Yumeng Dong, Mrinal K. Sarkar, Yuntian Wu, Xianying Xing, Matthew T. Patrick, Bo Duan, Mehrnaz Gharaee-Kermani, Valerie Julia, Stephan Weidinger, J. Michelle Kahlenberg, Johann E. Gudjonsson, Lam C. Tsoi

**Affiliations:** 1 Department of Dermatology, Michigan Medicine, Ann Arbor, Michigan, USA; 2 Department of Dermatology, Xiangya third Hospital, Central South University, Changsha, China; 3 Department of Biostatistics, School of Public Health, University of Michigan, Ann Arbor, Michigan, USA; 4 Department of Molecular, Cellular and Developmental Biology, University of Michigan, Ann Arbor, Michigan, USA; 5 Galderma, Lausanne, Switzerland; 6 Department of Dermatology and Allergy, University Medical Center Schleswig-Holstein, Campus Kiel, Germany; 7 Division of Rheumatology, Department of Internal Medicine, University of Michigan, Ann Arbor, Michigan, USA; 8 Taubman Medical Research Institute, Michigan Medicine, Ann Arbor, Michigan, USA; 9 Mary H. Weiser Food Allergy Center, Michigan Medicine, Ann Arbor, Michigan, USA; 10 Department of Computational Medicine & Bioinformatics, Michigan Medicine, Ann Arbor, Michigan, USA

**Keywords:** ET-2, IL-31, Itch, Keratinocytes, Prurigo nodularis

## TO THE EDITOR

In chronic skin diseases such as atopic dermatitis (AD) or prurigo nodularis (PN), interactions between keratinocytes, immune cells, and nerve endings exacerbate both epidermal damage and the sensation of pruritus. ET-1, encoded by *EDN1*, is a nonhistaminergic itch mediator that induces dose-dependent scratching in BALB/c mice and a burning itch in humans upon intradermal injection (Katugampola et al, 2000; McQueen et al, 2007). Elevated ET-1 levels are associated with increased disease severity in AD and psoriasis (Bonifati et al, 1998; Tsybikov et al, 2015). Despite the extensive research on ET-1, its isomer ET-2—encoded by *EDN2*—has been less studied (Ling et al, 2013). Both belong to the endothelin family and exhibit minor structural differences, primarily at the side chains of Trp6 and Leu7 (Davenport et al, 2016). They bind with equal affinity to 2 G-protein-coupled receptors, the ETA and ETB receptors (Davenport et al, 2016). Early research indicates that ET-2 in human skin mimics the actions of ET-1, suggesting a potential role in regulating itch, although investigations into its mechanisms are still limited (Katugampola et al, 2000; Ling et al, 2013).

**Figure 1.**
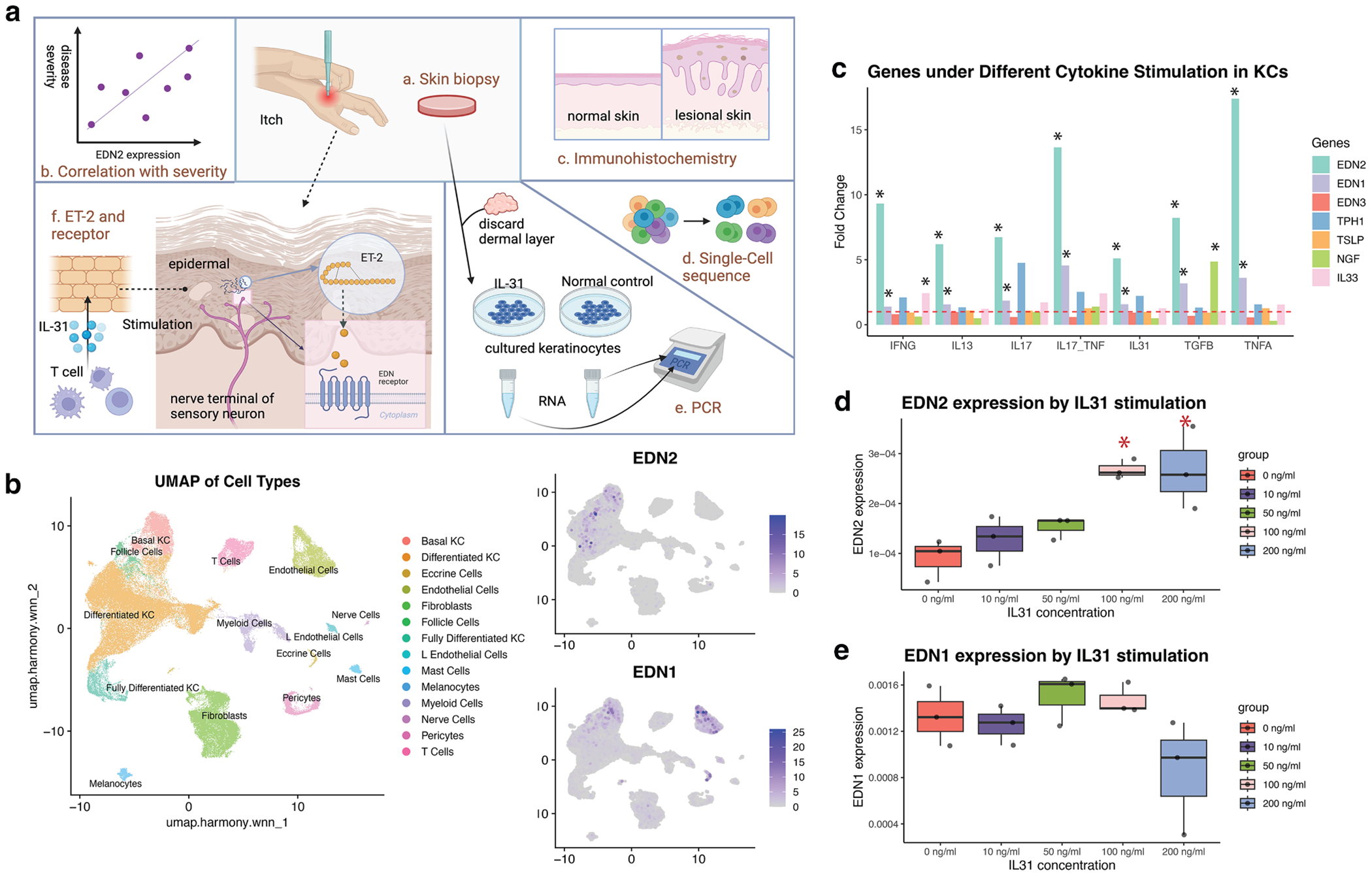
EDN2 is a keratinocytes-specific mediator stimulated by IL-31. (**a**) Our workflow began with collecting skin biopsies from patients, establishing correlations between gene expression and disease severity, quantifying protein levels through immunohistochemistry, analyzing cell-type distribution and expression differences using scRNA-seq, and isolating and culturing keratinocytes to assess RNA expression changes in response to specific stimuli through PCR, highlighting the interaction between *EDN2* expressed in keratinocytes and the *EDNRB* receptor on nerve endings to uncover mechanistic insights into itch-related responses. This image was created in BioRender.com. (**b**) *EDN1/EDN2* expression in different cell-type groups in normal skin, including basal keratinocytes (*K14*+), differentiated keratinocytes (*K10*+), and fully differentiated keratinocytes (*FLG*+). (**c**) Bar graph of fold changes in gene expression of mediators under various cytokine stimulations (marked * when FDR < 0.05). (**d**) *EDN2* expression under IL-31 gradient concentration stimulation (marked * when *P* < .05). (**e**) *EDN1* expression under IL-31 gradient concentration stimulation (marked * when *P* < .05). Expression levels are relative to RPLP0, calculated as 10(−**ΔΔ**Ct). FDR, false discovery rate; K10, keratin 10; K14, keratin 14; scRNA-seq, single-cell RNA-sequencing.

Figure 1a provides an overview of our study on the role of *EDN2* in itch. We utilized single-cell RNA sequencing to reveal that *EDN2* expression is keratinocyte specific. *EDN2* is expressed in the basal (keratin 14 gene *K14*+) and differentiated (keratin 10 gene *K10*+) keratinocytes, whereas *EDN1* is expressed primarily in endothelial cells and basal keratinocytes (Figure 1b). Keratinocytes respond to various cytokines by secreting mediators (eg, ET-1, TSLP, NGF, and IL-33) to induce pruritus (Misery et al, 2023). We profiled these mediators under primary keratinocytes stimulated with different cytokines (Figure 1c). We revealed that *EDN2* is induced with the largest magnitude than other itch mediators from keratinocytes. IL-31, a key itch mediator, significantly induced *EDN2* expression (fold change [FC] = 5.10, *P* = 6.3 × 10^−5^). We observed similar responses for other cytokine stimulation, with *EDN2* being upregulated by IL-13 (FC = 6.19, *P* = 2.0 × 10^−10^), TNF (FC = 17.39, *P* = 4.2 × 10^−14^), IL-17 (FC = 6.73, *P* = 5.0 × 10^−6^), IL-17 + TNF (FC = 13.64, *P* = 1.2 × 10^−10^), IFNg (FC = 9.32, *P* = 2.8 × 10^−7^), TGFβ1 (FC = 8.22, *P* = 4.4 × 10^−7^). On the contrary, *EDN1* exhibited a less pronounced and less significant pattern. We further confirmed these findings using N/TERT-2G (Dickson et al, 2000) cell lines, showing that IL-31 stimulation for *EDN2* expression has a dosage effect (Figure 1d), whereas no significant difference was observed for *EDN1* (Figure 1e).

**Figure 2.**
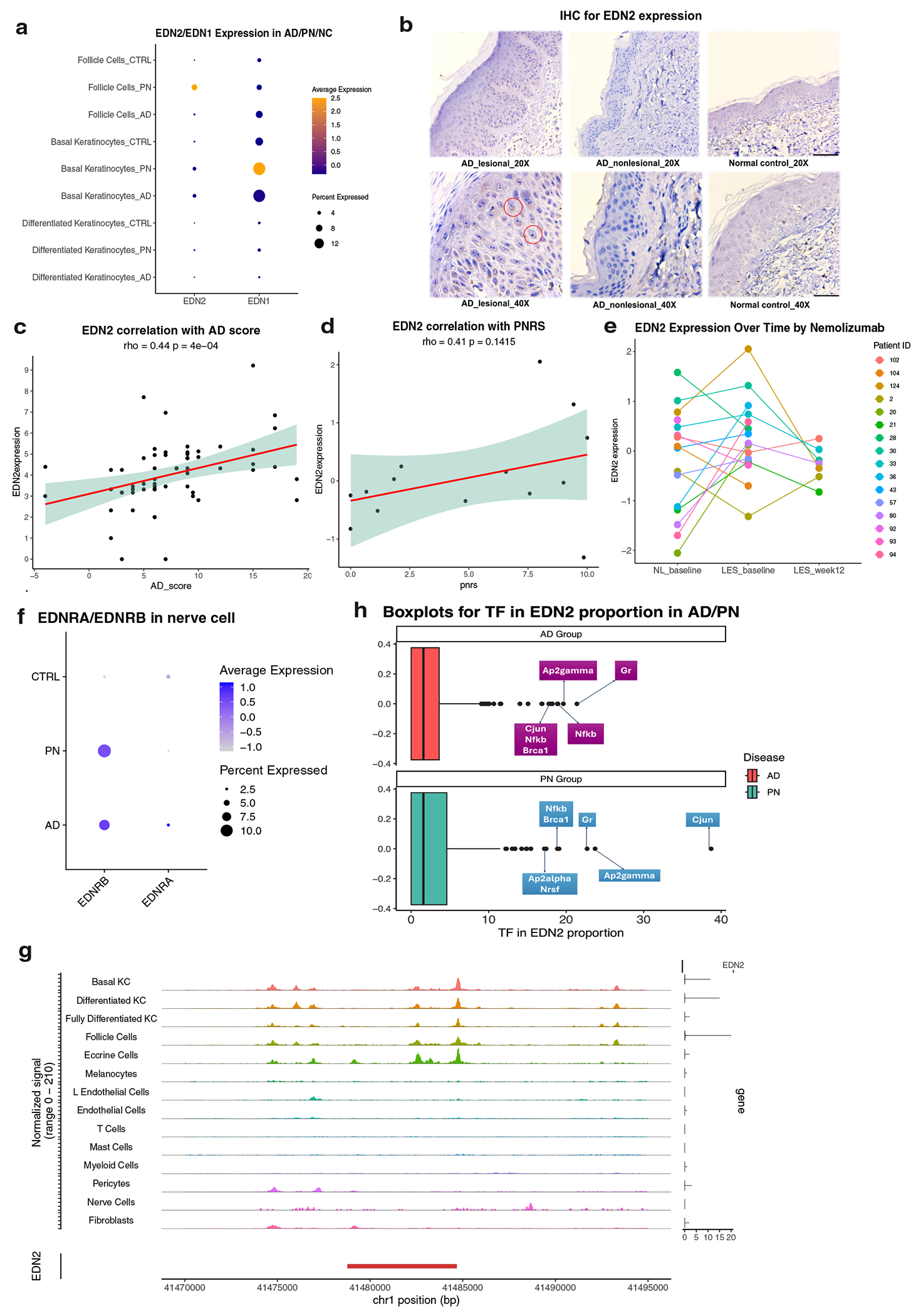
Expression of EDN2 is higher in lesional PN and AD skin and correlates with disease severity. (**a**) EDN*1* and EDN*2* expression in lesional skin of PN/AD compared with that in NC. (**b**) Immunohistochemistry of *EDN2* in AD lesional skin, nonlesional skin, and normal skin. The images were captured under a microscope at ×20 (bar = 200 μm) and ×40 (bar = 100 μm) magnification. (**c**) The correlation between AD severity score (SCORAD) and *EDN2* expression. (**d**) The correlation between PN severity score (PNRS) and *EDN2*. (**e**) *EDN2* expression in nonlesional, lesional, and 12-week nemolizumab-treated groups. (**f**) Expression of *EDNRA/EDNRB* in nerve cell. (**g**) Local ATAC peaks linked to expression of *EDN2.* (**h**) The proportion of TFs associated with *EDN2* peaks in AD/PN samples relative to the entire genome (the expected value is ~1). AD, atopic dermatitis; ATAC, Assay for Transposase-Accessible Chromatin; KC, keratinocyte; NC, normal control; PN, prurigo nodularis; PNRS, Peak Pruritus Numerical Rating Scale; SCORAD, SCORing Atopic Dermatitis; TF, transcription factor.

Utilizing single-cell RNA sequencing on skin of mice with PN and AD (Ma et al, 2024), we showed that *EDN2* has increased expression in hair follicle cells in lesional PN skin compared with that in the control (Figure 2a and Supplementary Table S1), and EDN1 showed a similar pattern. We performed immunohistochemistry staining to profile pro-ET2 in AD (Figure 2b) and PN (Supplementary Figure S1a) lesional skin. Our results confirmed that *EDN2* expression is increased in the epidermis of lesional AD and PN skin compared with that in healthy control skin.

We then quantified the relationship between *EDN2* expression and disease severity. Utilizing bulk RNA sequencing, we analyzed skin samples from 2 independent cohorts with AD and PN. A significant positive correlation was found between *EDN2* expression and SCORing Atopic Dermatitis on the basis of the severity of skin signs and patients’ itch intensity (Spearman’s correlation ρ = 0.44, *P* = 4 × 10^−4^) (Figure 2c). For PN, *EDN2* expression also showed a positive correlation with the Peak Pruritus Numerical Rating Scale (*ρ* = 0.41, *P* = .14) (Figure 2d); the lack of statistically significant differences can be attributed to a smaller sample size, and future studies with larger cohorts are needed to confirm this association. On the contrary, no positive correlation was identified for *EDN1* (Supplementary Figure S1b and c). Next, we studied the effect of anti—IL-31 receptor treatment on *EDN2* in the PN cohort. For the nemolizumab-treated group, 5 of the 7 patients with elevated *EDN2* expression in lesional skin at baseline (when comparing against nonlesional) exhibited lower expression after 12-week treatment (Figure 2e). In comparison, only 1 of 10 patients in the placebo-treated group showed the same pattern (Supplementary Figure S1d). We observed *EDN1* showing a more random expression pattern in the same analysis (Supplementary Figure S1e and f). These results indicate that *EDN2* is affected by the anti—IL-31 receptor treatment. We also conducted a similar analysis for *EDN3*, another gene in the endothelin family, and interestingly, it seemed to play a role in fibroblasts and was not elevated in lesional skin of patients with PN (Supplementary Figure S2). For the receptors of endothelin, we used single-cell RNA sequencing to reveal that *EDNRB* is expressed in nerve cells (refer to the nerve terminal of sensory neurons) (Supplementary Figure S1g) and shows increased expression in lesional skin from patients with AD and PN (Figure 2f) (FC_PN/control_ = 1.10, *P* = 2.1 × 10^−2^, FC_AD/control_ = 1.08, *P* = 4.1 × 10^−2^), whereas *EDNRA* was not differentially expressed.

To explore the mechanism of ET-2 regulation, we used Assay for Transposase-Accessible Chromatin with high-throughput sequencing from skin of patients with AD, patients with PN, and healthy individuals. We identified open chromatin regions within the ±250 k range surrounding the *EDN2* gene and applied DIRECT-NET (Zhang et al, 2022) to correlate accessibility with *EDN2* expression. We showed that the *EDN2* promoter region captured by the Assay for Transposase-Accessible Chromatin peaks is only present in keratinocytes, hair follicles, and eccrine cells (Figure 2g), concurrent with the findings mentioned earlier. We revealed 59 intergenic peaks linked to *EDN2*. We employed chromatin immunoprecipitation sequencing data from ENCODE to explore the potential transcription factors regulating *EDN2*. Figure 2h illustrates the proportion of transcription factors associated with *EDN2*-linked peaks in AD/PN samples relative to the genome. Transcription factors such as Cjun, Gr, AP2gamma, AP2alpha, and Nf-kB were found to have a higher proportion in the *EDN2*-linked peaks of both AD and PN samples. In IL-31—stimulated keratinocytes, some of these transcription factors are upregulated: JUN (encoding Cjun, FC = 1.42, *P* = 4.4 × 10^−3^), NFKB1 (encoding NFKB p50, FC = 1.24, *P* = 2.3 × 10^−2^), and NFKB2 (encoding NFKB p52, FC = 1.52, *P* = 4.3 × 10^−3^). Similar to *EDN2*, NFKB2 also showed upregulation in other cytokine stimulations, including IL-13 (FC = 1.47, *P* = 4.1 × 10^−2^), TNF (FC = 4, *P* = 2.7 × 10^−28^), IL-17 (FC = 1.66, *P* = 2.0 × 10^−3^), IL-17 + TNF (FC = 3.43, *P* = 6.1 × 10^−15^), IFNg (FC = 1.58, *P* = 1.0 × 10 ^−3^). Owing to the complex structure and broad functions of NFKB, the effects of NFKB2 on *EDN2* warrant more systematic and in-depth study.

Collectively, our findings position *EDN2* as a keratinocyte-derived biomarker of pruritus severity in AD and PN, with correlative evidence (eg, IL-31 responsiveness), suggesting its potential contribution to itch signaling. Although definitive mechanistic validation is needed, these data nominate *EDN2* as a priority candidate for future functional studies in chronic itch.

## Supplementary Material

1

Supplementary material is linked to the online version of the paper at www.jidonline.org, and at 10.1016/j.jid.2025.07.030.

## Data Availability

The single-cell RNA-sequencing data used in this manuscript are made available in Gene Expression Omnibus GSE273559 (https://www.ncbi.nlm.nih.gov/geo/query/acc.cgi?acc=GSE273559).

## Abbreviations:

AD: atopic dermatitis
FC: fold change
PN: prurigo nodularis

## References

[R1] Bonifati C, Mussi A, Carducci M, Pittarello A, D’Auria L, Venuti A, Endothelin-1 levels are increased in sera and lesional skin extracts of psoriatic patients and correlate with disease severity. Acta Derm Venereol 1998;78:22–6. 9498021 10.1080/00015559850135779

[R2] Davenport AP, Hyndman KA, Dhaun N, Southan C, Kohan DE, Pollock JS, Endothelin. Pharmacol Rev 2016;68:357–418. 26956245 10.1124/pr.115.011833 PMC4815360

[R3] Dickson MA, Hahn WC, Ino Y, Ronfard V, Wu JY, Weinberg RA, Human keratinocytes that express hTERT and also bypass a p16(INK4a)-enforced mechanism that limits life span become immortal yet retain normal growth and differentiation characteristics. Mol Cell Biol 2000;20:1436–47. 10648628 10.1128/mcb.20.4.1436-1447.2000 PMC85304

[R4] Katugampola R, Church MK, Clough GF. The neurogenic vasodilator response to endothelin-1: a study in human skin in vivo. Exp Physiol 2000;85:839–46. 11187978

[R5] Ling L, Maguire JJ, Davenport AP. Endothelin-2, the forgotten isoform: emerging role in the cardiovascular system, ovarian development, immunology and cancer. Br J Pharmacol 2013;168:283–95. 22118774 10.1111/j.1476-5381.2011.01786.x PMC3572556

[R6] Ma F, Gharaee-Kermani M, Tsoi LC, Plazyo O, Chaskar P, Harms P, Single-cell profiling of prurigo nodularis demonstrates immune-stromal crosstalk driving profibrotic responses and reversal with nemolizumab. J Allergy Clin Immunol 2024;153:146–60. 37506977 10.1016/j.jaci.2023.07.005 PMC11231883

[R7] McQueen DS, Noble MA, Bond SM. Endothelin-1 activates ETA receptors to cause reflex scratching in BALB/c mice. Br J Pharmacol 2007;151:278–84. 17351652 10.1038/sj.bjp.0707216 PMC2013956

[R8] Misery L, Pierre O, Le Gall-Ianotto C, Lebonvallet N, Chernyshov PV, Le Garrec R, Basic mechanisms of itch. J Allergy Clin Immunol 2023;152:11–23. 37201903 10.1016/j.jaci.2023.05.004

[R9] Tsybikov NN, Petrisheva IV, Kuznik BI, Magen E. Plasma endothelin-1 levels during exacerbation of atopic dermatitis. Allergy Asthma Proc 2015;36:320–4. 26108089 10.2500/aap.2015.36.3846

[R10] Zhang L, Zhang J, Nie Q. DIRECT-NET: an efficient method to discover cis-regulatory elements and construct regulatory networks from single-cell multiomics data. Sci Adv 2022;8:eabl7393. 35648859 10.1126/sciadv.abl7393 PMC9159696

