# Supplementary material for: Endothelin-2 from Keratinocytes and its Association with Itch in Skin Diseases
Source: J Invest Dermatol. Author manuscript; Available in PMC 2026 Jun 23. (PMC13289584; doi:10.1016/j.jid.2025.07.030)
Supplement: 1 [file NIHMS2181700-supplement-1.pdf]

SUPPLEMENTARY MATERIALS AND METHODS

Profiling *EDN2* and *EDN1* from cytokine-stimulated keratinocytes

Primary keratinocytes were collected from healthy donors' 6-mm skin biopsy samples. We used 0.4% Dispase II (Gibco, Thermo Fisher Scientific, catalog number 17105041) overnight to separate the epidermal and dermal layers and then cultured the epidermis in the Keratinocyte Serum-Free Medium (KSFM) (GIBCO, 0.09 mM calcium ion, catalog number 17005042). Five keratinocyte samples were stimulated with cytokines IL-31 (R&D Systems, 10 ng/ml, catalog number 10425-IL-020), IL-13 (R&D Systems, 10 ng/ml, catalog number 213-ILB-005), IL-17A (R&D Systems, 10 ng/ml, catalog number 314-ILB-050), TNFα (R&D Systems, 10 ng/ml, catalog number 210-TA-005), IFNa (R&D Systems, 5 ng/ml, catalog number 11100-1), IFNg (R&D Systems, 5 ng/ml, catalog number 285-IF-100), and TFGb (R&D Systems, 10 ng/ml, catalog number 240-B-002). Cytokines were grouped on the basis of their roles and associations with T helper cell subsets. IL-36 and IL-1β were analyzed for T helper 1 responses; IL-4 and IL-13 were categorized under T helper 2 for their roles in allergic and antiparasitic responses; and the T helper 17 group included IL-17, both alone and combined with TNF (IL-17 + TNF). We conducted bulk RNA sequencing for each group. After adapter trimming, we conducted alignment to map reads to the human reference genome (hg19) using STAR (Dobin et al, 2013). Only uniquely mapped reads were used in the analysis. Gene quantification was performed using HTSeq (Anders et al, 2015). This study was reviewed and approved by the Michigan Medicine Institutional Review Board of the Medical School (HUM00151834).

Single-cell genomics and bulk RNA sequencing

Single-cell RNA-sequencing data were obtained from a prurigo nodularis/atopic dermatitis cohort with 6 patients with prurigo nodularis , 6 patients with atopic dermatitis , and 15 healthy donors, as previously detailed (Ma et al, 2024), and we also utilized a skin single-cell RNA-sequencing dataset consisting of 96 control samples (https://doi.org/10.25452/figshare.plus.25696620.v1). For bulk RNA sequencing, we employed an atopic dermatitis cohort with SCORing Atopic Dermatitis to measure disease severity (Tsoi et al, 2020) as well as a prurigo nodularis cohort with Peak Pruritus Numerical Rating Scale to measure disease severity and tracked the changes in Peak Pruritus Numerical Rating Scale over 12 weeks in patients treated with nemolizumab and a placebo group (Tsoi et al, 2022). All differential expression *P*-values from both bulk and single-cell analyses were adjusted for multiple testing using the Benjamini−Hochberg procedure to control the false discovery rate.

We conducted single nuclei multiome from 4-mm healthy individuals' skin biopsies. Biopsies were incubated in 0.4% Dispase II (Gibco, Thermo Fisher Scientific, catalog number 17105041) overnight to separate the epidermis and dermis. After the separation, the epidermis was transferred to a 0.25% Trypsin-EDTA + 10 units/ml DNase mixture and incubated at 37 °C for 1 hour. The epidermis mixture was then quenched with fetal bovine serum and precipitated by centrifugation. Cell pellets were then resuspended in PBS + 0.04% BSA. Cell numbers were counted at this step for future dilution calculation. Cells were harvested and subjected to transposition using the Tn5 transposase, which fragments DNA and simultaneously inserts sequencing adapters into accessible chromatin regions. After transposition, DNA was purified and then amplified through PCR to enrich tagged fragments and incorporate sequencing indices. The amplified library was purified, was quantified, and then underwent sequencing.

Alignment to the hg38 genome and initial processing were performed using 10X Genomics' Cell Ranger, and the resulting expression and peak matrices were merged without depth normalization. All downstream analyses were carried out in Seurat (version 4.3.0) and Signac (version 1.9.0). Ambient RNA contamination was then corrected with SoupX, and barcode multiplets were detected and removed with scDblFinder. Nuclei with nucleosome signal ≥4, transcription start sites enrichment ≤2, or mitochondrial gene fraction ≥25% were excluded. Normalization was performed using term-frequency inverse-document-frequency and singular value decomposition with default parameters for the peak assay. Finally, *cis*-regulatory elements within ±250 kb of the EDN2 transcription start site were linked to EDN2 expression using the gradient-boosting framework DIRECT-NET (Zhang et al, 2022).

IL-31−stimulated keratinocytes cell line

We cultured N/TERT-2G cell line (Dickson et al, 2000) in Keratinocyte SFM (1X) (Thermo Fisher Scientific, catalog number 17005042), supplemented with human keratinocyte growth supplement (Gibco, catalog number S0015) and bovine pituitary extract (Gibco, catalog number 13028014), using a gradient of IL-31(recombinant human IL-31 [HEK293-expressed] protein, R&D Systems, catalog number 10425-IL-020), that is 0, 10, 50, 100, and 200 ng/ml, to stimulate for 24 hours. Each well starts with 50,000 cells, with 3 replicates per group. After treatment, we extracted RNA (RNeasy Kits, Qiagen, catalog number 74104), performed reverse transcription (TaqMan, catalog number 4366596), and conducted qRT-PCR. *RPLP0* (TaqMan primer) was used as the housekeeping gene. EDN2 primer from Taqman.

Immunohistochemistry

We selected 3 paired lesional and nonlesional skin samples from both patients with prurigo nodularis and those with atopic dermatitis, along with 3 normal skin samples. The samples were embedded in paraffin and then sectioned into thin slices, which were placed onto glass slides. The slides with tissue sections were then heated at 60 °C for 30 minutes, followed by immersion in xylene and sequential immersion in an ethanol gradient (100, 100, 95, 85, and 75%). The slides were put in retrieval buffer (pH = 6, BD Biosciences) under the pressure cooker water bath for 30 seconds for antigen retrieval. After retrieval, the slides were blocked with serum and subsequently incubated with an EDN2 primary antibody (Sigma, number HPA028459, 1:50 dilution) at 4 °C overnight. The

next day, the slides were washed with PBS with Tween and treated with a secondary antibody (1:200, Rabbit Anti-Goat IgG Antibody, Vector Laboratories), followed by washes. Then, horseradish peroxidase (ABC Kits, Vector Laboratories) was added for 30 minutes. Development was achieved using 3,3′-diaminobenzidine for exactly 3.5 minutes under microscope monitoring. The sections were then counterstained with hematoxylin, dehydrated, and mounted. Microscopic examination and photography were performed to evaluate the staining results.

SUPPLEMENTARY REFERENCES

Anders S, Pyl PT, Huber W. HTSeq–a Python framework to work with high-throughput sequencing data. Bioinformatics 2015;31:166–9.

Dickson MA, Hahn WC, Ino Y, Ronfard V, Wu JY, Weinberg RA, et al. Human keratinocytes that express hTERT and also bypass a p16(INK4a)-enforced mechanism that limits life span become immortal yet retain normal growth and differentiation characteristics. Mol Cell Biol 2000;20:1436–47.

Dobin A, Davis CA, Schlesinger F, Drenkow J, Zaleski C, Jha S, et al. STAR: ultrafast universal RNA-seq aligner. Bioinformatics 2013;29:15–21.

Ma F, Gharaee-Kermani M, Tsoi LC, Plazyo O, Chaskar P, Harms P, et al. Single-cell profiling of prurigo nodularis demonstrates immune-stromal crosstalk driving profibrotic responses and reversal with nemolizumab. J Allergy Clin Immunol 2024;153:146–60.

Tsoi LC, Hacini-Rachinel F, Fogel P, Rousseau F, Xing X, Patrick MT, et al. Transcriptomic characterization of prurigo nodularis and the therapeutic response to nemolizumab. J Allergy Clin Immunol 2022;149:1329–39.

Tsoi LC, Rodriguez E, Stölzl D, Wehkamp U, Sun J, Gerdes S, et al. Progression of acute-to-chronic atopic dermatitis is associated with quantitative rather than qualitative changes in cytokine responses. J Allergy Clin Immunol 2020;145:1406–15.

Zhang L, Zhang J, Nie Q. DIRECT-NET: an efficient method to discover cis-regulatory elements and construct regulatory networks from single-cell multiomics data. Sci Adv 2022;8:eabl7393.

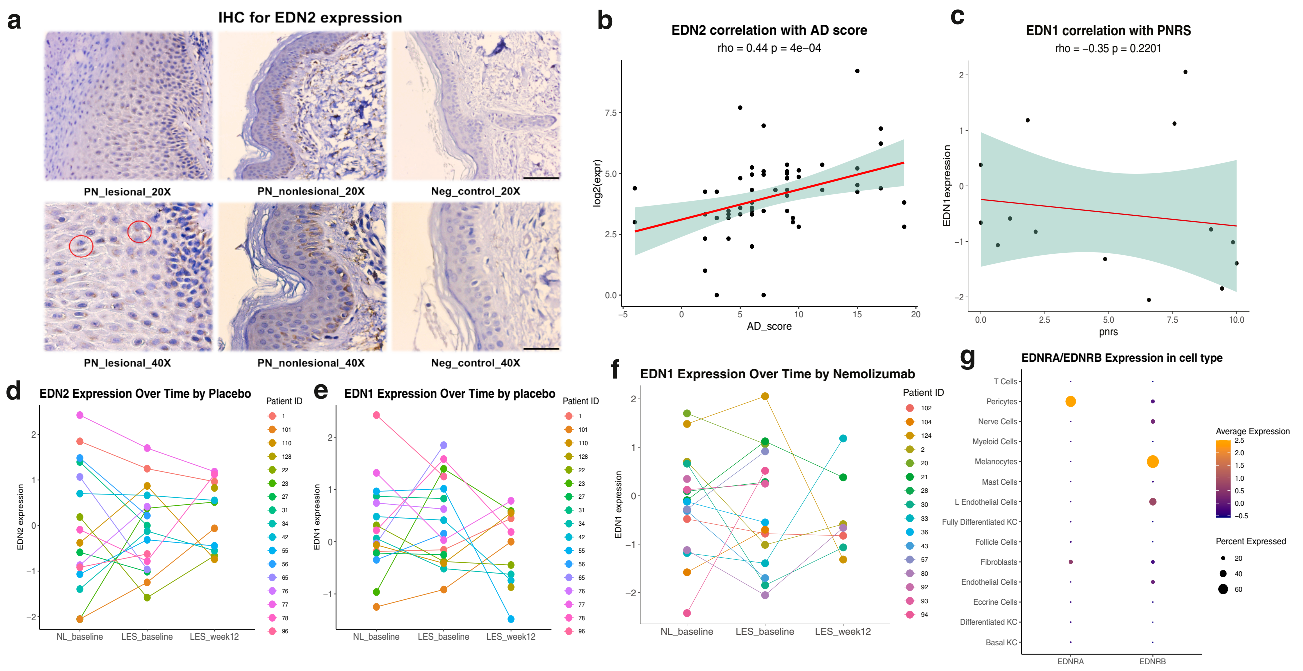

Supplementary Figure S1. Expression patterns of *EDN2* and *EDN1*. (**a**) Immunohistochemistry of *EDN2* in PN lesional skin, nonlesional skin, and negative control. The images were captured under a microscope at ×20 (bar = 200 μm) and ×40 (bar = 100 μm) magnification. (**b**) The correlation between AD severity score and *EDN1* expression. (**c**) The correlation between PN severity score and *EDN1* expression. (**d**) *EDN2* expression in nonlesional, lesional, and 12-week placebo-treated groups. (**e, f**) *EDN1* expression for nonlesional and lesional at baseline and lesional in week 12 for (**e**) placebo and (**f**) treatment groups. (**g**) EDNRA/EDNRB expression in cell types of normal skin. AD, atopic dermatitis; PN, prurigo nodularis.

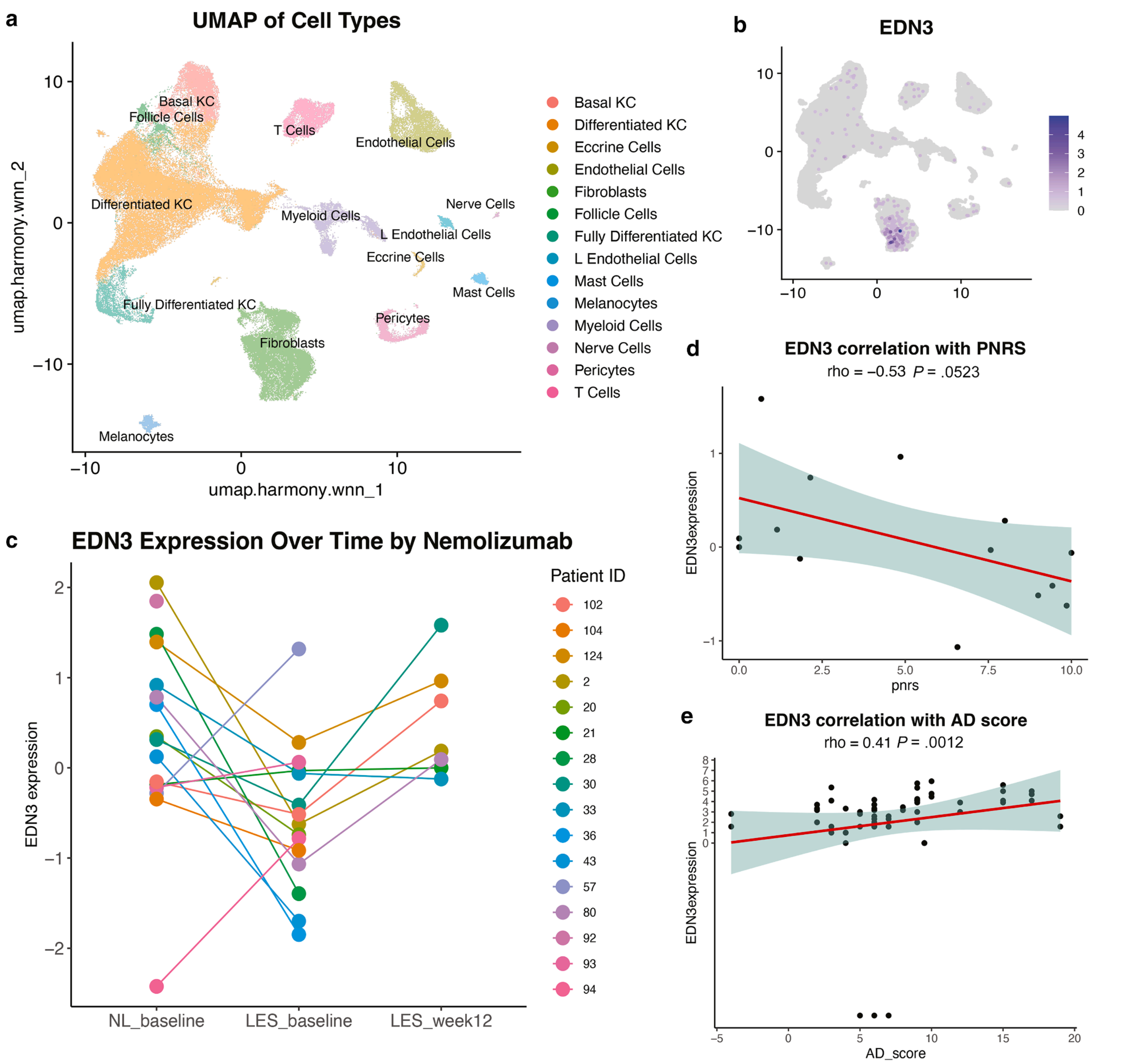

Supplementary Figure S2. Expression of *EDN3*. (**a, b**) *EDN3* expression in different cell types from scRNA-seq. (**c**) *EDN3* expression in nonlesional, lesional, and 12-week nemolizumab-treated groups. (**d**) The correlation between PN severity score (PNRS) and *EDN3*. (**e**) The correlation between AD severity score (SCORAD) and *EDN3* expression. PN, prurigo nodularis; PNRS, Peak Pruritus Numerical Rating Scale; SCORAD, SCORing Atopic Dermatitis; scRNA-seq, single-cell RNA sequencing.

Supplementary Table S1. Expression Changes of *EDN2* and *EDN1* in Lesional Skin Versus CTRL Skin in Different Cell Types

Gene	Cell Type	$FC_{PN/CTRL}$	$P_{PN/CTRL}$	$FC_{AD/CTRL}$	$P_{AD/CTRL}$
EDN2	Follicle cell	1.15	1.8×10^{-4}	1.01	.29
	Basal cell	1.94	1.4×10^{-5}	1.05	8.5×10^{-6}
EDN1	Follicle cell	1.04	.26	1.07	2.6×10^{-2}
	Basal cell	1.11	6.1×10^{-7}	1.10	7.1×10^{-8}

Abbreviations: AD, atopic dermatitis; CTRL, control; FC, fold change; PN, prurigo nodularis.
